# A Nonsurgical Treatise on the Diseases of the Prostate Gland and Adnexa

**Published:** 1907-08

**Authors:** 


**A Nonsurgical Treatise on the Diseases of the Prostate Gland and Adnexa.** By **George Whitfield Overall,** A.B., M.D. 12 mo. pp. 228, Illustrated. Rowe Publishing Co. 1906.

If one were to give rein to one's intimate fancies and consider this work as a novelty, it might be brought into that literary fold where are gathered the outputs of faddists, and to do that, would be charitable. But one needs must be serious at times, at least. However, why draw on one's reserve fund of charity when it might be better saved for a more worthy purpose. It would take the mantle of a charity organisation society to cover the sins of this book. It has one virtue—it is brief; hence not much time is consumed in tripping lightly through its pages. There are glimmering points scattered through the pages of the book which approach art; yet the thing which most appeals to one is that it is kaleidoscopic; there is science, art, some literary construction, heavy and light reading and a flavor of fiction.

